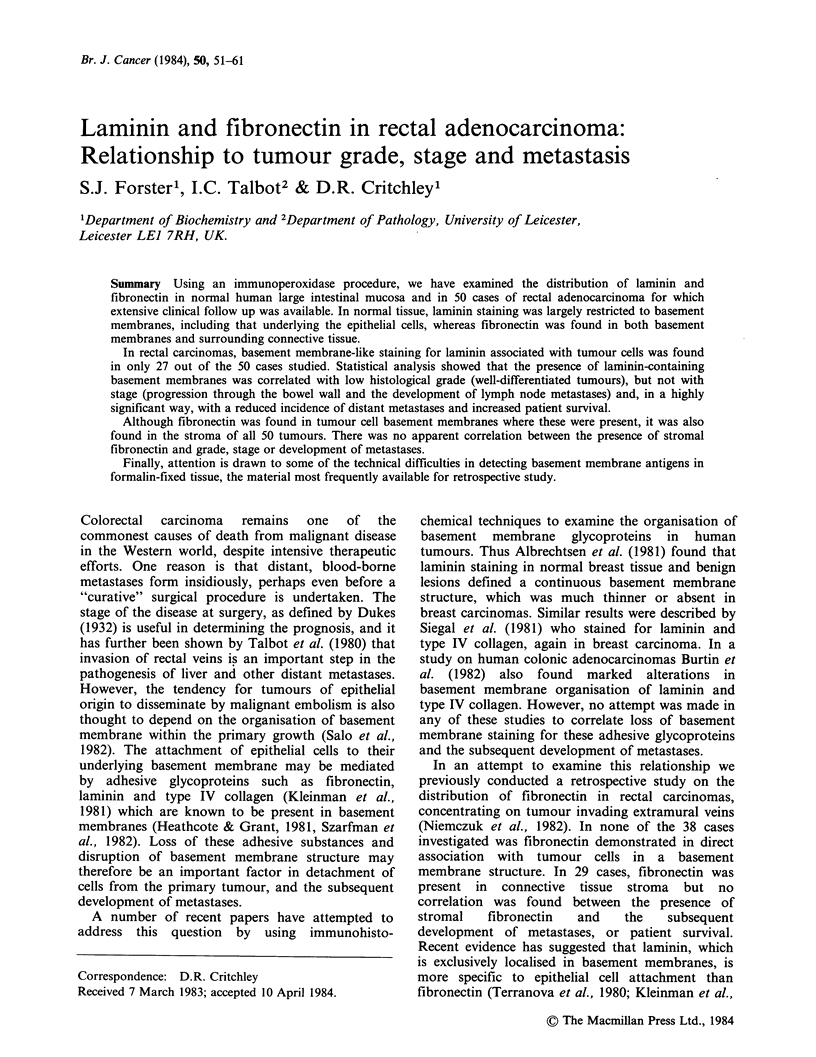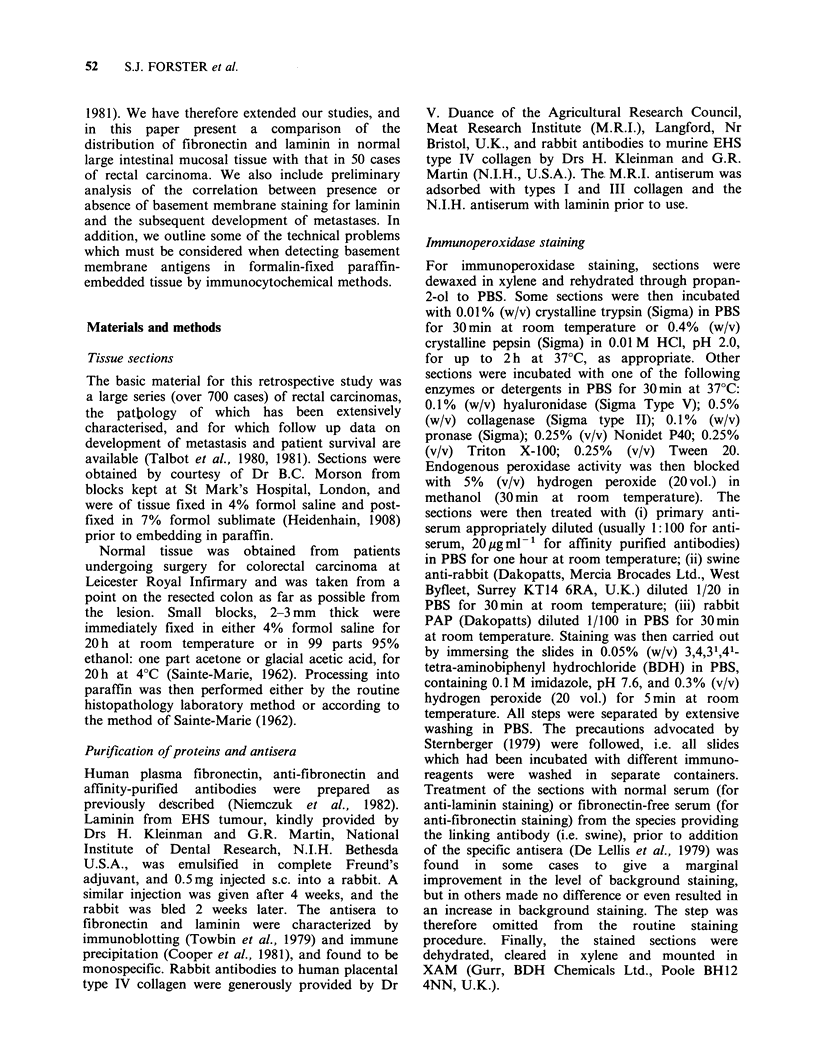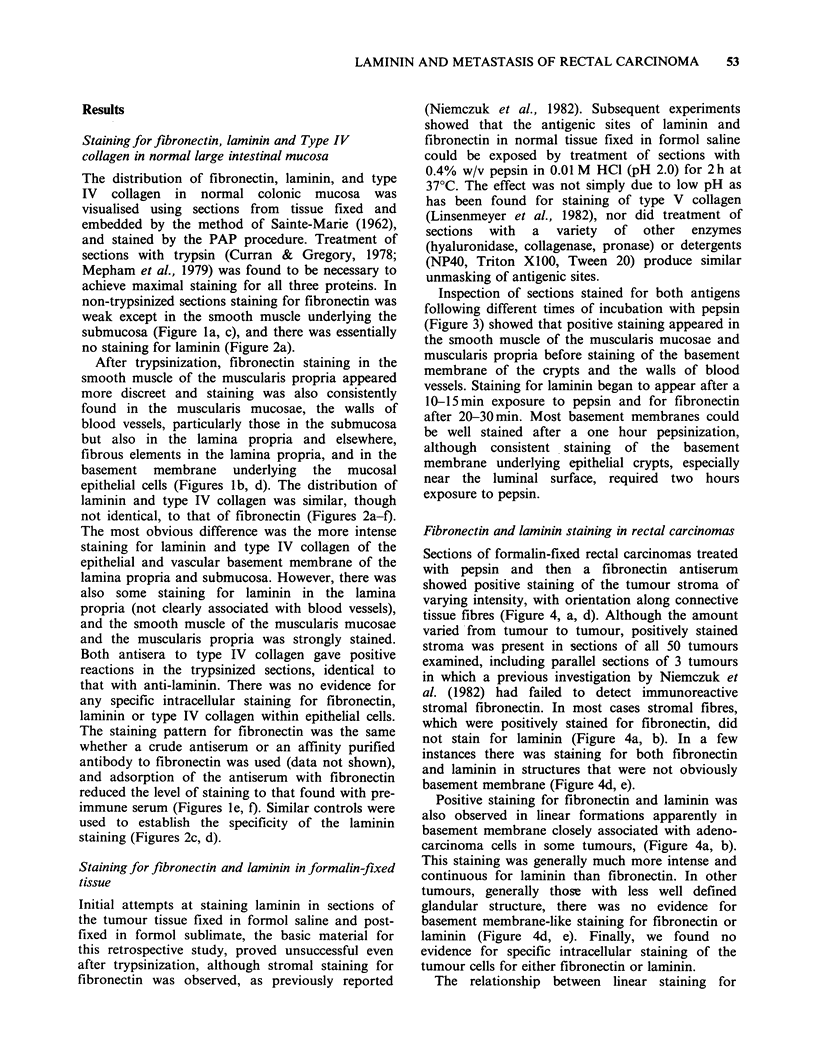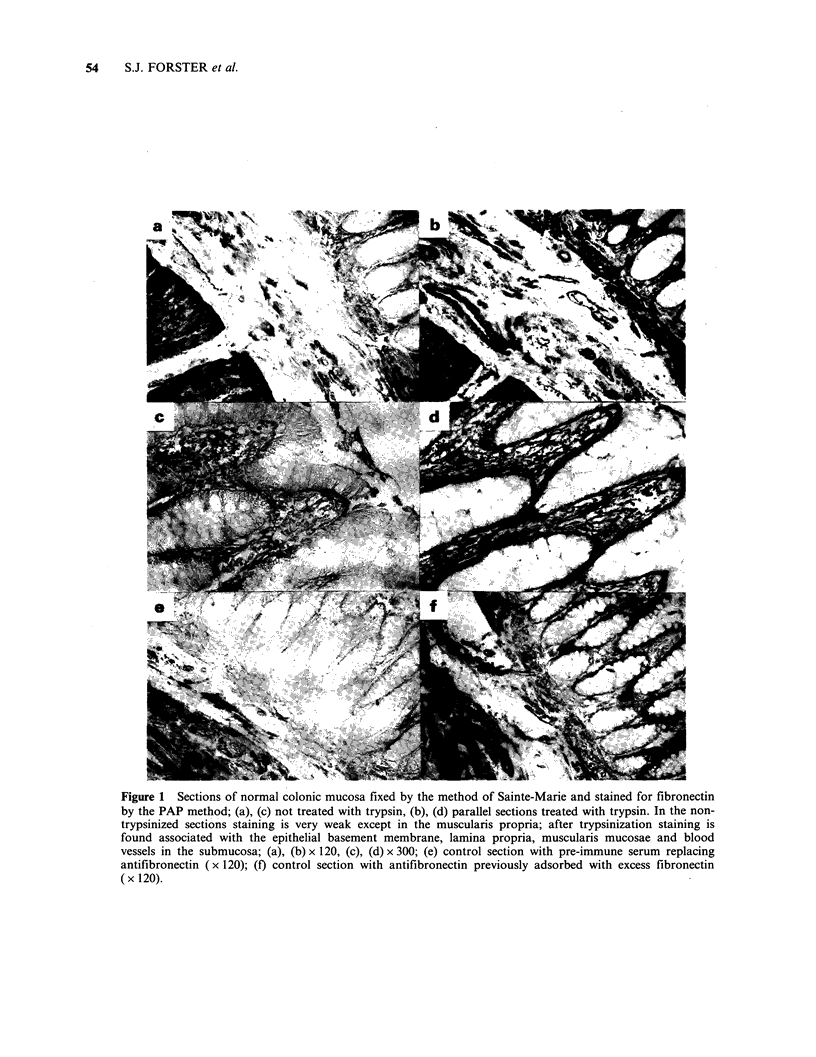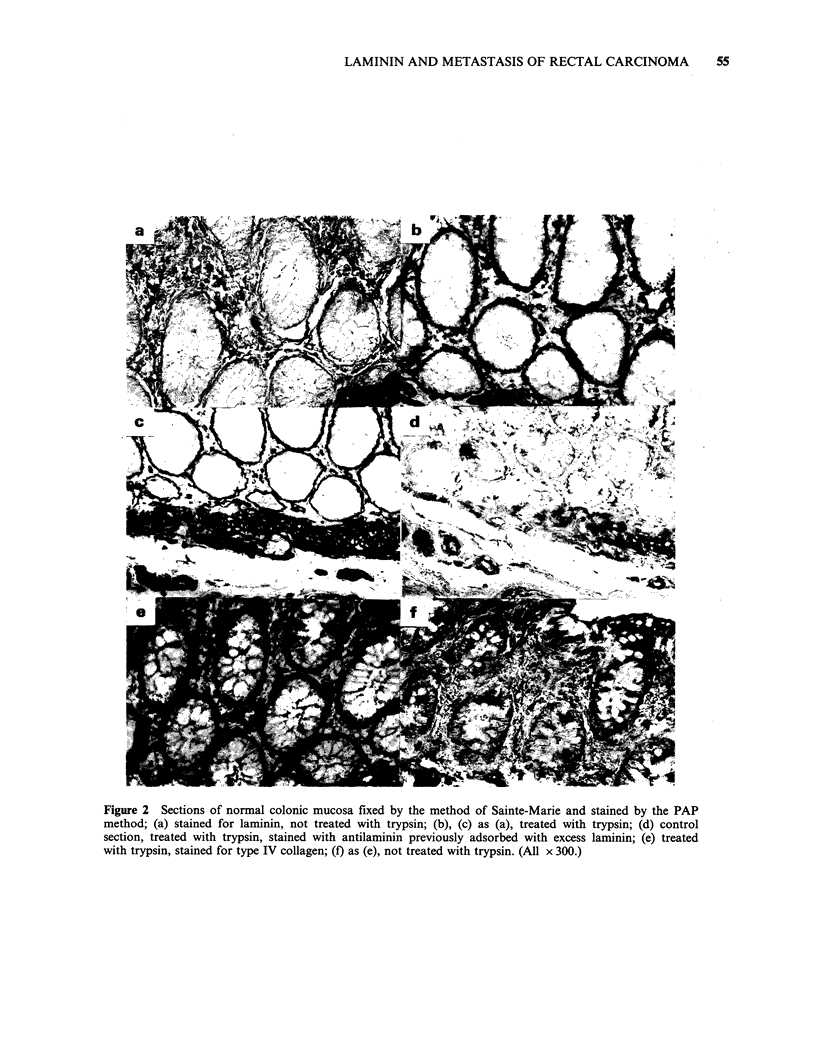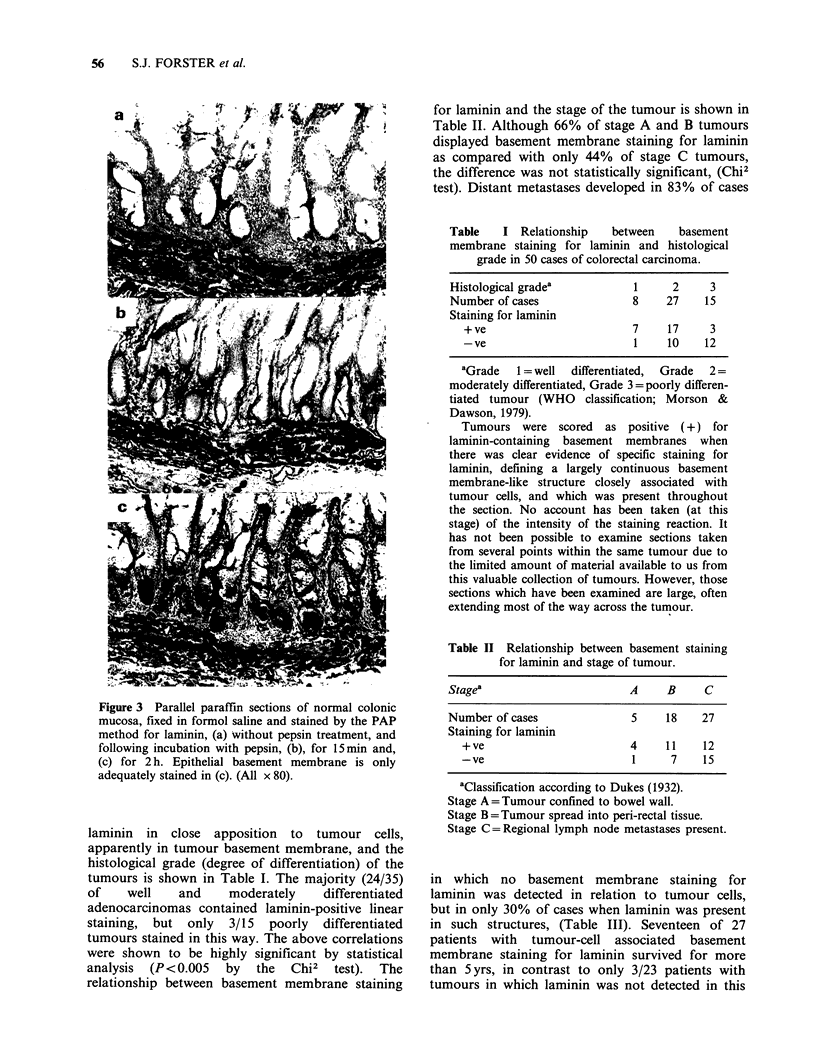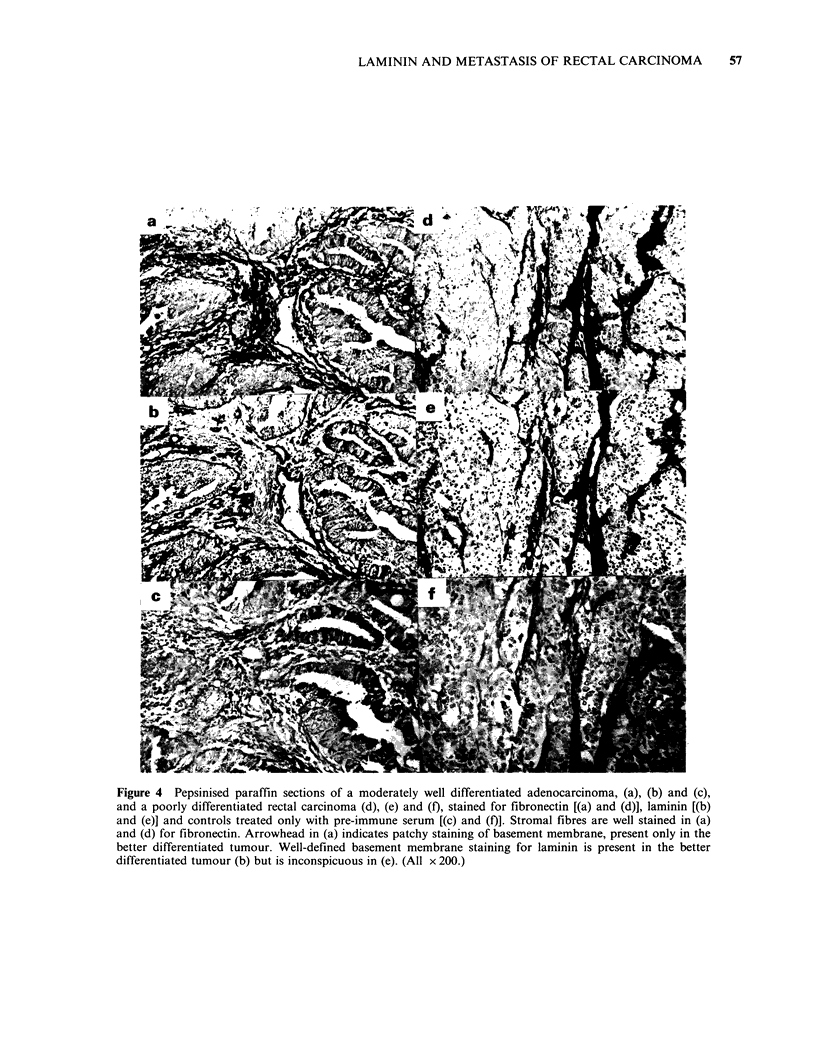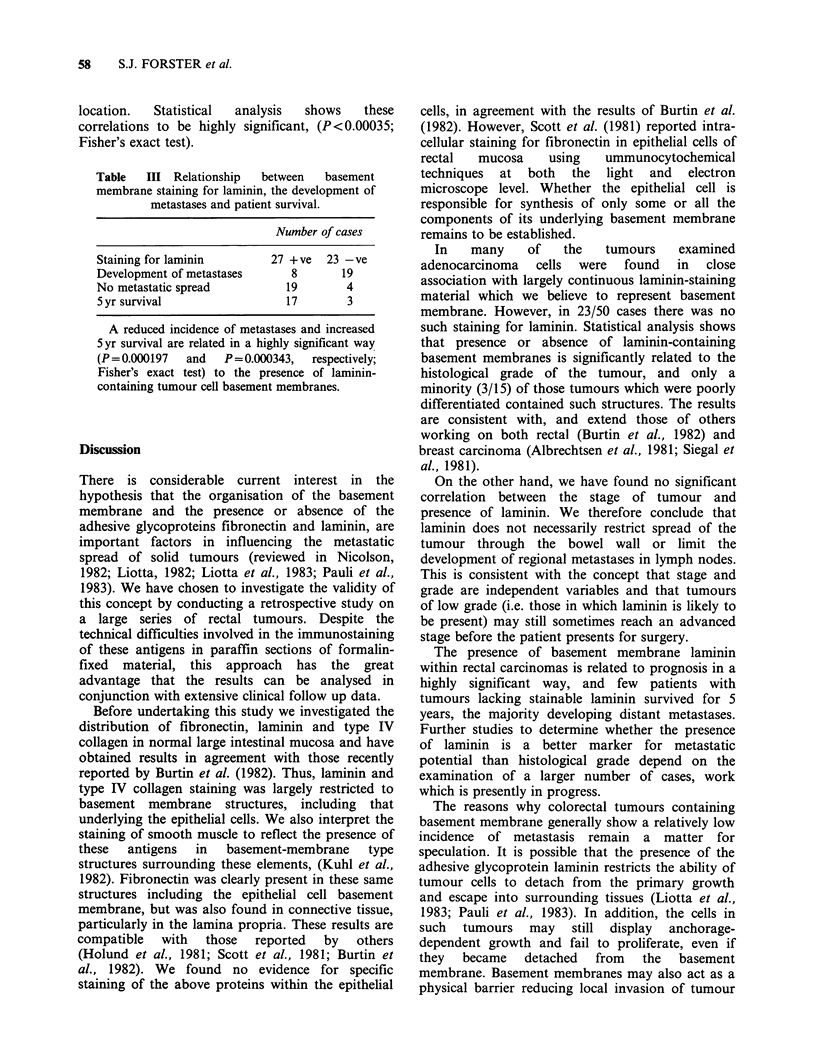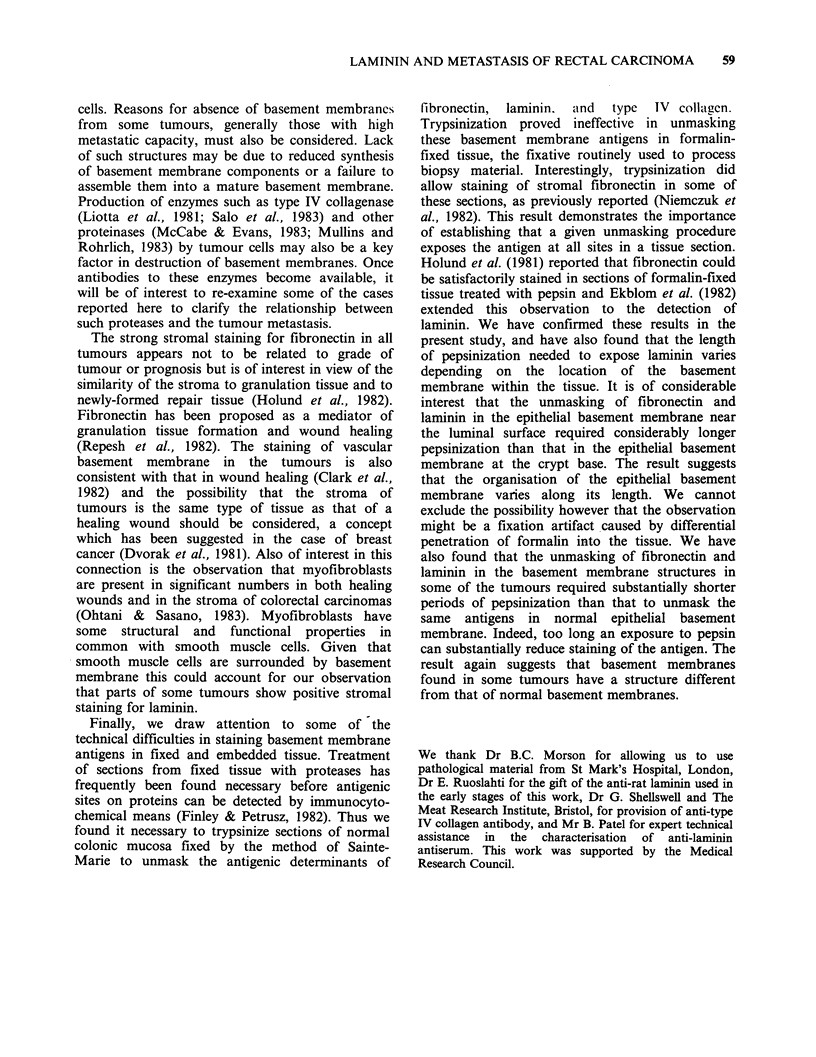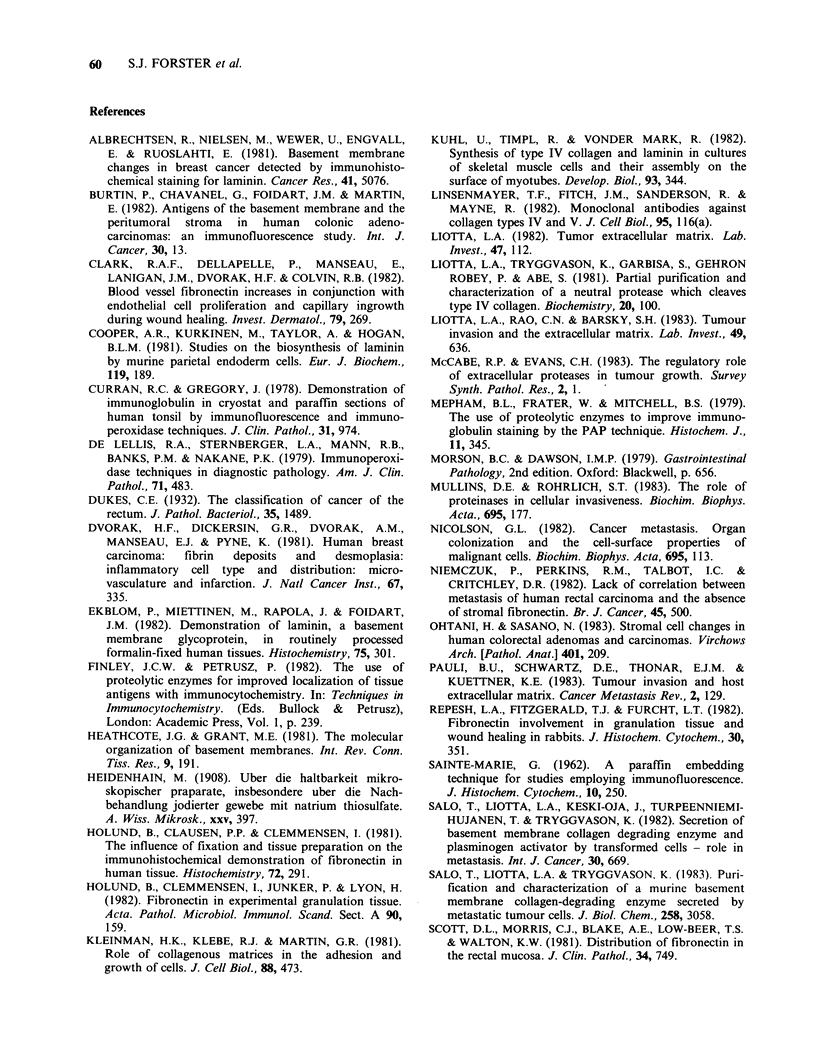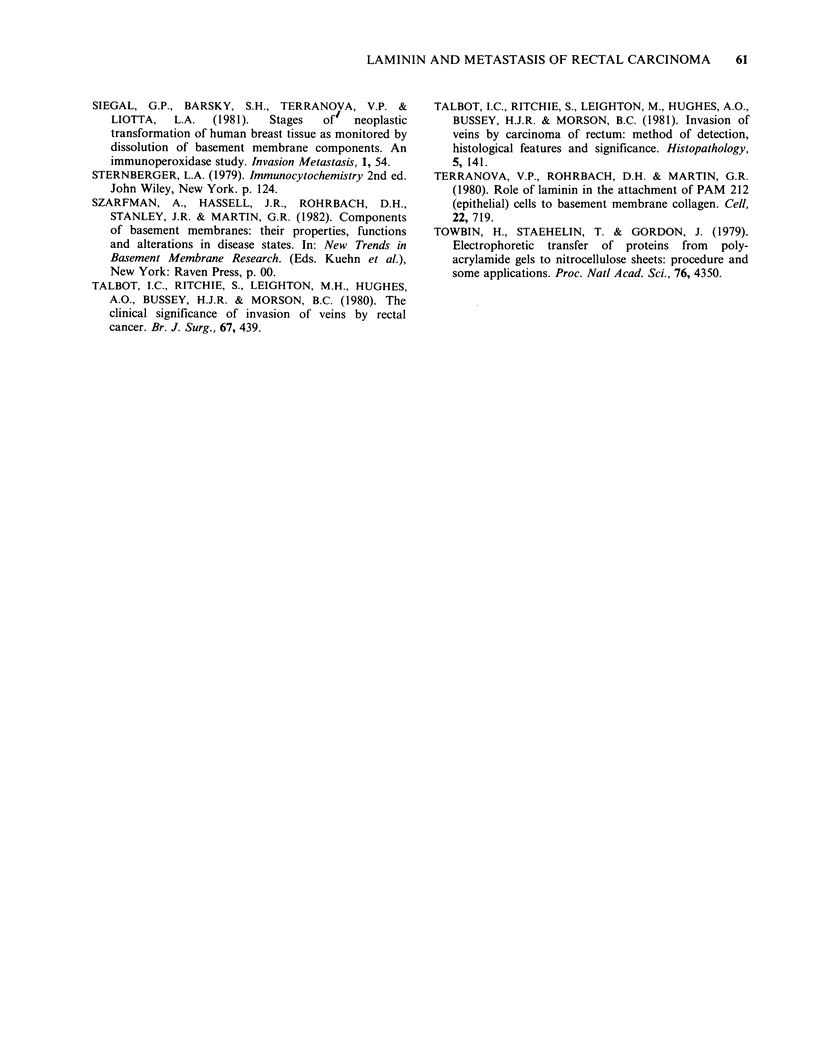# Laminin and fibronectin in rectal adenocarcinoma: relationship to tumour grade, stage and metastasis.

**DOI:** 10.1038/bjc.1984.139

**Published:** 1984-07

**Authors:** S. J. Forster, I. C. Talbot, D. R. Critchley

## Abstract

**Images:**


*Br. J. Cancer* (1984), **50**, 51–61

### Laminin and fibronectin in rectal adenocarcinoma: Relationship to tumour grade, stage and metastasis

S.J. Forster[1], I.C. Talbot[2] & D.R. Critchley[1]

[1]*Department of Biochemistry and* [2]*Department of Pathology, University of Leicester, Leicester LE1 7RH, UK.*

**Summary** Using an immunoperoxidase procedure, we have examined the distribution of laminin and fibronectin in normal human large intestinal mucosa and in 50 cases of rectal adenocarcinoma for which extensive clinical follow up was available. In normal tissue, laminin staining was largely restricted to basement membranes, including that underlying the epithelial cells, whereas fibronectin was found in both basement membranes and surrounding connective tissue.

In rectal carcinomas, basement membrane-like staining for laminin associated with tumour cells was found in only 27 out of the 50 cases studied. Statistical analysis showed that the presence of laminin-containing basement membranes was correlated with low histological grade (well-differentiated tumours), but not with stage (progression through the bowel wall and the development of lymph node metastases) and, in a highly significant way, with a reduced incidence of distant metastases and increased patient survival.

Although fibronectin was found in tumour cell basement membranes where these were present, it was also found in the stroma of all 50 tumours. There was no apparent correlation between the presence of stromal fibronectin and grade, stage or development of metastases.

Finally, attention is drawn to some of the technical difficulties in detecting basement membrane antigens in formalin-fixed tissue, the material most frequently available for retrospective study.

Colorectal carcinoma remains one of the commonest causes of death from malignant disease in the Western world, despite intensive therapeutic efforts. One reason is that distant, blood-borne metastases form insidiously, perhaps even before a "curative" surgical procedure is undertaken. The stage of the disease at surgery, as defined by Dukes (1932) is useful in determining the prognosis, and it has further been shown by Talbot *et al.* (1980) that invasion of rectal veins is an important step in the pathogenesis of liver and other distant metastases. However, the tendency for tumours of epithelial origin to disseminate by malignant embolism is also thought to depend on the organisation of basement membrane within the primary growth (Salo *et al.*, 1982). The attachment of epithelial cells to their underlying basement membrane may be mediated by adhesive glycoproteins such as fibronectin, laminin and type IV collagen (Kleinman *et al.*, 1981) which are known to be present in basement membranes (Heathcote & Grant, 1981, Szarfman *et al.*, 1982). Loss of these adhesive substances and disruption of basement membrane structure may therefore be an important factor in detachment of cells from the primary tumour, and the subsequent development of metastases.

A number of recent papers have attempted to address this question by using immunohistochemical techniques to examine the organisation of basement membrane glycoproteins in human tumours. Thus Albrechtsen *et al.* (1981) found that laminin staining in normal breast tissue and benign lesions defined a continuous basement membrane structure, which was much thinner or absent in breast carcinomas. Similar results were described by Siegal *et al.* (1981) who stained for laminin and type IV collagen, again in breast carcinoma. In a study on human colonic adenocarcinomas Burtin *et al.* (1982) also found marked alterations in basement membrane organisation of laminin and type IV collagen. However, no attempt was made in any of these studies to correlate loss of basement membrane staining for these adhesive glycoproteins and the subsequent development of metastases.

In an attempt to examine this relationship we previously conducted a retrospective study on the distribution of fibronectin in rectal carcinomas, concentrating on tumour invading extramural veins (Niemczuk *et al.*, 1982). In none of the 38 cases investigated was fibronectin demonstrated in direct association with tumour cells in a basement membrane structure. In 29 cases, fibronectin was present in connective tissue stroma but no correlation was found between the presence of stromal fibronectin and the subsequent development of metastases, or patient survival. Recent evidence has suggested that laminin, which is exclusively localised in basement membranes, is more specific to epithelial cell attachment than fibronectin (Terranova *et al.*, 1980; Kleinman *et al.*,

Correspondence: D.R. Critchley
Received 7 March 1983; accepted 10 April 1984.

© The Macmillan Press Ltd., 1984

1981). We have therefore extended our studies, and in this paper present a comparison of the distribution of fibronectin and laminin in normal large intestinal mucosal tissue with that in 50 cases of rectal carcinoma. We also include preliminary analysis of the correlation between presence or absence of basement membrane staining for laminin and the subsequent development of metastases. In addition, we outline some of the technical problems which must be considered when detecting basement membrane antigens in formalin-fixed paraffin-embedded tissue by immunocytochemical methods.

### Materials and methods

### *Tissue sections*

The basic material for this retrospective study was a large series (over 700 cases) of rectal carcinomas, the pathology of which has been extensively characterised, and for which follow up data on development of metastasis and patient survival are available (Talbot *et al.*, 1980, 1981). Sections were obtained by courtesy of Dr B.C. Morson from blocks kept at St Mark's Hospital, London, and were of tissue fixed in 4% formol saline and post-fixed in 7% formol sublimate (Heidenhain, 1908) prior to embedding in paraffin.

Normal tissue was obtained from patients undergoing surgery for colorectal carcinoma at Leicester Royal Infirmary and was taken from a point on the resected colon as far as possible from the lesion. Small blocks, 2–3 mm thick were immediately fixed in either 4% formol saline for 20 h at room temperature or in 99 parts 95% ethanol: one part acetone or glacial acetic acid, for 20 h at 4°C (Sainte-Marie, 1962). Processing into paraffin was then performed either by the routine histopathology laboratory method or according to the method of Sainte-Marie (1962).

### *Purification of proteins and antisera*

Human plasma fibronectin, anti-fibronectin and affinity-purified antibodies were prepared as previously described (Niemczuk *et al.*, 1982). Laminin from EHS tumour, kindly provided by Drs H. Kleinman and G.R. Martin, National Institute of Dental Research, N.I.H. Bethesda U.S.A., was emulsified in complete Freund's adjuvant, and 0.5 mg injected s.c. into a rabbit. A similar injection was given after 4 weeks, and the rabbit was bled 2 weeks later. The antisera to fibronectin and laminin were characterized by immunoblotting (Towbin *et al.*, 1979) and immune precipitation (Cooper *et al.*, 1981), and found to be monospecific. Rabbit antibodies to human placental type IV collagen were generously provided by Dr V. Duance of the Agricultural Research Council, Meat Research Institute (M.R.I.), Langford, Nr Bristol, U.K., and rabbit antibodies to murine EHS type IV collagen by Drs H. Kleinman and G.R. Martin (N.I.H., U.S.A.). The M.R.I. antiserum was adsorbed with types I and III collagen and the N.I.H. antiserum with laminin prior to use.

### *Immunoperoxidase staining*

For immunoperoxidase staining, sections were dewaxed in xylene and rehydrated through propan-2-ol to PBS. Some sections were then incubated with 0.01% (w/v) crystalline trypsin (Sigma) in PBS for 30 min at room temperature or 0.4% (w/v) crystalline pepsin (Sigma) in 0.01 M HCl, pH 2.0, for up to 2 h at 37°C, as appropriate. Other sections were incubated with one of the following enzymes or detergents in PBS for 30 min at 37°C: 0.1% (w/v) hyaluronidase (Sigma Type V); 0.5% (w/v) collagenase (Sigma type II); 0.1% (w/v) pronase (Sigma); 0.25% (v/v) Nonidet P40; 0.25% (v/v) Triton X-100; 0.25% (v/v) Tween 20. Endogenous peroxidase activity was then blocked with 5% (v/v) hydrogen peroxide (20 vol.) in methanol (30 min at room temperature). The sections were then treated with (i) primary anti-serum appropriately diluted (usually 1:100 for anti-serum, 20 $\mu g\,ml^{-1}$ for affinity purified antibodies) in PBS for one hour at room temperature; (ii) swine anti-rabbit (Dakopatts, Mercia Brocades Ltd., West Byfleet, Surrey KT14 6RA, U.K.) diluted 1/20 in PBS for 30 min at room temperature; (iii) rabbit PAP (Dakopatts) diluted 1/100 in PBS for 30 min at room temperature. Staining was then carried out by immersing the slides in 0.05% (w/v) $3,4,3^1,4^1$-tetra-aminobiphenyl hydrochloride (BDH) in PBS, containing 0.1 M imidazole, pH 7.6, and 0.3% (v/v) hydrogen peroxide (20 vol.) for 5 min at room temperature. All steps were separated by extensive washing in PBS. The precautions advocated by Sternberger (1979) were followed, i.e. all slides which had been incubated with different immuno-reagents were washed in separate containers. Treatment of the sections with normal serum (for anti-laminin staining) or fibronectin-free serum (for anti-fibronectin staining) from the species providing the linking antibody (i.e. swine), prior to addition of the specific antisera (De Lellis *et al.*, 1979) was found in some cases to give a marginal improvement in the level of background staining, but in others made no difference or even resulted in an increase in background staining. The step was therefore omitted from the routine staining procedure. Finally, the stained sections were dehydrated, cleared in xylene and mounted in XAM (Gurr, BDH Chemicals Ltd., Poole BH12 4NN, U.K.).

### Results

### *Staining for fibronectin, laminin and Type IV collagen in normal large intestinal mucosa*

The distribution of fibronectin, laminin, and type IV collagen in normal colonic mucosa was visualised using sections from tissue fixed and embedded by the method of Sainte-Marie (1962), and stained by the PAP procedure. Treatment of sections with trypsin (Curran & Gregory, 1978; Mepham *et al.*, 1979) was found to be necessary to achieve maximal staining for all three proteins. In non-trypsinized sections staining for fibronectin was weak except in the smooth muscle underlying the submucosa (Figure 1a, c), and there was essentially no staining for laminin (Figure 2a).

After trypsinization, fibronectin staining in the smooth muscle of the muscularis propria appeared more discreet and staining was also consistently found in the muscularis mucosae, the walls of blood vessels, particularly those in the submucosa but also in the lamina propria and elsewhere, fibrous elements in the lamina propria, and in the basement membrane underlying the mucosal epithelial cells (Figures 1b, d). The distribution of laminin and type IV collagen was similar, though not identical, to that of fibronectin (Figures 2a–f). The most obvious difference was the more intense staining for laminin and type IV collagen of the epithelial and vascular basement membrane of the lamina propria and submucosa. However, there was also some staining for laminin in the lamina propria (not clearly associated with blood vessels), and the smooth muscle of the muscularis mucosae and the muscularis propria was strongly stained. Both antisera to type IV collagen gave positive reactions in the trypsinized sections, identical to that with anti-laminin. There was no evidence for any specific intracellular staining for fibronectin, laminin or type IV collagen within epithelial cells. The staining pattern for fibronectin was the same whether a crude antiserum or an affinity purified antibody to fibronectin was used (data not shown), and adsorption of the antiserum with fibronectin reduced the level of staining to that found with pre-immune serum (Figures 1e, f). Similar controls were used to establish the specificity of the laminin staining (Figures 2c, d).

### *Staining for fibronectin and laminin in formalin-fixed tissue*

Initial attempts at staining laminin in sections of the tumour tissue fixed in formol saline and post-fixed in formol sublimate, the basic material for this retrospective study, proved unsuccessful even after trypsinization, although stromal staining for fibronectin was observed, as previously reported (Niemczuk *et al.*, 1982). Subsequent experiments showed that the antigenic sites of laminin and fibronectin in normal tissue fixed in formol saline could be exposed by treatment of sections with 0.4% w/v pepsin in 0.01 M HCl (pH 2.0) for 2 h at 37°C. The effect was not simply due to low pH as has been found for staining of type V collagen (Linsenmeyer *et al.*, 1982), nor did treatment of sections with a variety of other enzymes (hyaluronidase, collagenase, pronase) or detergents (NP40, Triton X100, Tween 20) produce similar unmasking of antigenic sites.

Inspection of sections stained for both antigens following different times of incubation with pepsin (Figure 3) showed that positive staining appeared in the smooth muscle of the muscularis mucosae and muscularis propria before staining of the basement membrane of the crypts and the walls of blood vessels. Staining for laminin began to appear after a 10–15 min exposure to pepsin and for fibronectin after 20–30 min. Most basement membranes could be well stained after a one hour pepsinization, although consistent staining of the basement membrane underlying epithelial crypts, especially near the luminal surface, required two hours exposure to pepsin.

### *Fibronectin and laminin staining in rectal carcinomas*

Sections of formalin-fixed rectal carcinomas treated with pepsin and then a fibronectin antiserum showed positive staining of the tumour stroma of varying intensity, with orientation along connective tissue fibres (Figure 4, a, d). Although the amount varied from tumour to tumour, positively stained stroma was present in sections of all 50 tumours examined, including parallel sections of 3 tumours in which a previous investigation by Niemczuk *et al.* (1982) had failed to detect immunoreactive stromal fibronectin. In most cases stromal fibres, which were positively stained for fibronectin, did not stain for laminin (Figure 4a, b). In a few instances there was staining for both fibronectin and laminin in structures that were not obviously basement membrane (Figure 4d, e).

Positive staining for fibronectin and laminin was also observed in linear formations apparently in basement membrane closely associated with adenocarcinoma cells in some tumours, (Figure 4a, b). This staining was generally much more intense and continuous for laminin than fibronectin. In other tumours, generally those with less well defined glandular structure, there was no evidence for basement membrane-like staining for fibronectin or laminin (Figure 4d, e). Finally, we found no evidence for specific intracellular staining of the tumour cells for either fibronectin or laminin.

The relationship between linear staining for

**Figure 1** Sections of normal colonic mucosa fixed by the method of Sainte-Marie and stained for fibronectin by the PAP method; (a), (c) not treated with trypsin, (b), (d) parallel sections treated with trypsin. In the non-trypsinized sections staining is very weak except in the muscularis propria; after trypsinization staining is found associated with the epithelial basement membrane, lamina propria, muscularis mucosae and blood vessels in the submucosa; (a), (b) × 120, (c), (d) × 300; (e) control section with pre-immune serum replacing antifibronectin ( × 120); (f) control section with antifibronectin previously adsorbed with excess fibronectin ( × 120).

**Figure 2** Sections of normal colonic mucosa fixed by the method of Sainte-Marie and stained by the PAP method; (a) stained for laminin, not treated with trypsin; (b), (c) as (a), treated with trypsin; (d) control section, treated with trypsin, stained with antilaminin previously adsorbed with excess laminin; (e) treated with trypsin, stained for type IV collagen; (f) as (e), not treated with trypsin. (All ×300.)

**Figure 3** Parallel paraffin sections of normal colonic mucosa, fixed in formol saline and stained by the PAP method for laminin, (a) without pepsin treatment, and following incubation with pepsin, (b), for 15 min and, (c) for 2 h. Epithelial basement membrane is only adequately stained in (c). (All ×80).

laminin in close apposition to tumour cells, apparently in tumour basement membrane, and the histological grade (degree of differentiation) of the tumours is shown in Table I. The majority (24/35) of well and moderately differentiated adenocarcinomas contained laminin-positive linear staining, but only 3/15 poorly differentiated tumours stained in this way. The above correlations were shown to be highly significant by statistical analysis ($P<0.005$ by the Chi$^2$ test). The relationship between basement membrane staining for laminin and the stage of the tumour is shown in Table II. Although 66% of stage A and B tumours displayed basement membrane staining for laminin as compared with only 44% of stage C tumours, the difference was not statistically significant, (Chi$^2$ test). Distant metastases developed in 83% of cases in which no basement membrane staining for laminin was detected in relation to tumour cells, but in only 30% of cases when laminin was present in such structures, (Table III). Seventeen of 27 patients with tumour-cell associated basement membrane staining for laminin survived for more than 5 yrs, in contrast to only 3/23 patients with tumours in which laminin was not detected in this

**Table I** Relationship between basement membrane staining for laminin and histological grade in 50 cases of colorectal carcinoma.

| | | | |
|---|---|---|---|
| Histological grade[a] | 1 | 2 | 3 |
| Number of cases | 8 | 27 | 15 |
| Staining for laminin | | | |
| +ve | 7 | 17 | 3 |
| −ve | 1 | 10 | 12 |

[a]Grade 1 = well differentiated, Grade 2 = moderately differentiated, Grade 3 = poorly differentiated tumour (WHO classification; Morson & Dawson, 1979).

Tumours were scored as positive (+) for laminin-containing basement membranes when there was clear evidence of specific staining for laminin, defining a largely continuous basement membrane-like structure closely associated with tumour cells, and which was present throughout the section. No account has been taken (at this stage) of the intensity of the staining reaction. It has not been possible to examine sections taken from several points within the same tumour due to the limited amount of material available to us from this valuable collection of tumours. However, those sections which have been examined are large, often extending most of the way across the tumour.

**Table II** Relationship between basement staining for laminin and stage of tumour.

| *Stage*[a] | *A* | *B* | *C* |
|---|---|---|---|
| Number of cases | 5 | 18 | 27 |
| Staining for laminin | | | |
| +ve | 4 | 11 | 12 |
| −ve | 1 | 7 | 15 |

[a]Classification according to Dukes (1932).
Stage A = Tumour confined to bowel wall.
Stage B = Tumour spread into peri-rectal tissue.
Stage C = Regional lymph node metastases present.

**Figure 4** Pepsinised paraffin sections of a moderately well differentiated adenocarcinoma, (a), (b) and (c), and a poorly differentiated rectal carcinoma (d), (e) and (f), stained for fibronectin [(a) and (d)], laminin [(b) and (e)] and controls treated only with pre-immune serum [(c) and (f)]. Stromal fibres are well stained in (a) and (d) for fibronectin. Arrowhead in (a) indicates patchy staining of basement membrane, present only in the better differentiated tumour. Well-defined basement membrane staining for laminin is present in the better differentiated tumour (b) but is inconspicuous in (e). (All ×200.)

location. Statistical analysis shows these correlations to be highly significant, ($P<0.00035$; Fisher's exact test).

**Table III** Relationship between basement membrane staining for laminin, the development of metastases and patient survival.

| | Number of cases | |
|---|---|---|
| Staining for laminin | 27 +ve | 23 −ve |
| Development of metastases | 8 | 19 |
| No metastatic spread | 19 | 4 |
| 5 yr survival | 17 | 3 |

A reduced incidence of metastases and increased 5 yr survival are related in a highly significant way ($P=0.000197$ and $P=0.000343$, respectively; Fisher's exact test) to the presence of laminin-containing tumour cell basement membranes.

### Discussion

There is considerable current interest in the hypothesis that the organisation of the basement membrane and the presence or absence of the adhesive glycoproteins fibronectin and laminin, are important factors in influencing the metastatic spread of solid tumours (reviewed in Nicolson, 1982; Liotta, 1982; Liotta *et al.*, 1983; Pauli *et al.*, 1983). We have chosen to investigate the validity of this concept by conducting a retrospective study on a large series of rectal tumours. Despite the technical difficulties involved in the immunostaining of these antigens in paraffin sections of formalin-fixed material, this approach has the great advantage that the results can be analysed in conjunction with extensive clinical follow up data.

Before undertaking this study we investigated the distribution of fibronectin, laminin and type IV collagen in normal large intestinal mucosa and have obtained results in agreement with those recently reported by Burtin *et al.* (1982). Thus, laminin and type IV collagen staining was largely restricted to basement membrane structures, including that underlying the epithelial cells. We also interpret the staining of smooth muscle to reflect the presence of these antigens in basement-membrane type structures surrounding these elements, (Kuhl *et al.*, 1982). Fibronectin was clearly present in these same structures including the epithelial cell basement membrane, but was also found in connective tissue, particularly in the lamina propria. These results are compatible with those reported by others (Holund *et al.*, 1981; Scott *et al.*, 1981; Burtin *et al.*, 1982). We found no evidence for specific staining of the above proteins within the epithelial cells, in agreement with the results of Burtin *et al.* (1982). However, Scott *et al.* (1981) reported intra-cellular staining for fibronectin in epithelial cells of rectal mucosa using ummunocytochemical techniques at both the light and electron microscope level. Whether the epithelial cell is responsible for synthesis of only some or all the components of its underlying basement membrane remains to be established.

In many of the tumours examined adenocarcinoma cells were found in close association with largely continuous laminin-staining material which we believe to represent basement membrane. However, in 23/50 cases there was no such staining for laminin. Statistical analysis shows that presence or absence of laminin-containing basement membranes is significantly related to the histological grade of the tumour, and only a minority (3/15) of those tumours which were poorly differentiated contained such structures. The results are consistent with, and extend those of others working on both rectal (Burtin *et al.*, 1982) and breast carcinoma (Albrechtsen *et al.*, 1981; Siegal *et al.*, 1981).

On the other hand, we have found no significant correlation between the stage of tumour and presence of laminin. We therefore conclude that laminin does not necessarily restrict spread of the tumour through the bowel wall or limit the development of regional metastases in lymph nodes. This is consistent with the concept that stage and grade are independent variables and that tumours of low grade (i.e. those in which laminin is likely to be present) may still sometimes reach an advanced stage before the patient presents for surgery.

The presence of basement membrane laminin within rectal carcinomas is related to prognosis in a highly significant way, and few patients with tumours lacking stainable laminin survived for 5 years, the majority developing distant metastases. Further studies to determine whether the presence of laminin is a better marker for metastatic potential than histological grade depend on the examination of a larger number of cases, work which is presently in progress.

The reasons why colorectal tumours containing basement membrane generally show a relatively low incidence of metastasis remain a matter for speculation. It is possible that the presence of the adhesive glycoprotein laminin restricts the ability of tumour cells to detach from the primary growth and escape into surrounding tissues (Liotta *et al.*, 1983; Pauli *et al.*, 1983). In addition, the cells in such tumours may still display anchorage-dependent growth and fail to proliferate, even if they became detached from the basement membrane. Basement membranes may also act as a physical barrier reducing local invasion of tumour cells. Reasons for absence of basement membranes from some tumours, generally those with high metastatic capacity, must also be considered. Lack of such structures may be due to reduced synthesis of basement membrane components or a failure to assemble them into a mature basement membrane. Production of enzymes such as type IV collagenase (Liotta *et al.*, 1981; Salo *et al.*, 1983) and other proteinases (McCabe & Evans, 1983; Mullins and Rohrlich, 1983) by tumour cells may also be a key factor in destruction of basement membranes. Once antibodies to these enzymes become available, it will be of interest to re-examine some of the cases reported here to clarify the relationship between such proteases and the tumour metastasis.

The strong stromal staining for fibronectin in all tumours appears not to be related to grade of tumour or prognosis but is of interest in view of the similarity of the stroma to granulation tissue and to newly-formed repair tissue (Holund *et al.*, 1982). Fibronectin has been proposed as a mediator of granulation tissue formation and wound healing (Repesh *et al.*, 1982). The staining of vascular basement membrane in the tumours is also consistent with that in wound healing (Clark *et al.*, 1982) and the possibility that the stroma of tumours is the same type of tissue as that of a healing wound should be considered, a concept which has been suggested in the case of breast cancer (Dvorak *et al.*, 1981). Also of interest in this connection is the observation that myofibroblasts are present in significant numbers in both healing wounds and in the stroma of colorectal carcinomas (Ohtani & Sasano, 1983). Myofibroblasts have some structural and functional properties in common with smooth muscle cells. Given that smooth muscle cells are surrounded by basement membrane this could account for our observation that parts of some tumours show positive stromal staining for laminin.

Finally, we draw attention to some of the technical difficulties in staining basement membrane antigens in fixed and embedded tissue. Treatment of sections from fixed tissue with proteases has frequently been found necessary before antigenic sites on proteins can be detected by immunocytochemical means (Finley & Petrusz, 1982). Thus we found it necessary to trypsinize sections of normal colonic mucosa fixed by the method of Sainte-Marie to unmask the antigenic determinants of fibronectin, laminin, and type IV collagen. Trypsinization proved ineffective in unmasking these basement membrane antigens in formalin-fixed tissue, the fixative routinely used to process biopsy material. Interestingly, trypsinization did allow staining of stromal fibronectin in some of these sections, as previously reported (Niemczuk *et al.*, 1982). This result demonstrates the importance of establishing that a given unmasking procedure exposes the antigen at all sites in a tissue section. Holund *et al.* (1981) reported that fibronectin could be satisfactorily stained in sections of formalin-fixed tissue treated with pepsin and Ekblom *et al.* (1982) extended this observation to the detection of laminin. We have confirmed these results in the present study, and have also found that the length of pepsinization needed to expose laminin varies depending on the location of the basement membrane within the tissue. It is of considerable interest that the unmasking of fibronectin and laminin in the epithelial basement membrane near the luminal surface required considerably longer pepsinization than that in the epithelial basement membrane at the crypt base. The result suggests that the organisation of the epithelial basement membrane varies along its length. We cannot exclude the possibility however that the observation might be a fixation artifact caused by differential penetration of formalin into the tissue. We have also found that the unmasking of fibronectin and laminin in the basement membrane structures in some of the tumours required substantially shorter periods of pepsinization than that to unmask the same antigens in normal epithelial basement membrane. Indeed, too long an exposure to pepsin can substantially reduce staining of the antigen. The result again suggests that basement membranes found in some tumours have a structure different from that of normal basement membranes.

We thank Dr B.C. Morson for allowing us to use pathological material from St Mark's Hospital, London, Dr E. Ruoslahti for the gift of the anti-rat laminin used in the early stages of this work, Dr G. Shellswell and The Meat Research Institute, Bristol, for provision of anti-type IV collagen antibody, and Mr B. Patel for expert technical assistance in the characterisation of anti-laminin antiserum. This work was supported by the Medical Research Council.

### References

ALBRECHTSEN, R., NIELSEN, M., WEWER, U., ENGVALL, E. & RUOSLAHTI, E. (1981). Basement membrane changes in breast cancer detected by immunohistochemical staining for laminin. *Cancer Res.*, **41**, 5076.

BURTIN, P., CHAVANEL, G., FOIDART, J.M. & MARTIN, E. (1982). Antigens of the basement membrane and the peritumoral stroma in human colonic adenocarcinomas: an immunofluorescence study. *Int. J. Cancer*, **30**, 13.

CLARK, R.A.F., DELLAPELLE, P., MANSEAU, E., LANIGAN, J.M., DVORAK, H.F. & COLVIN, R.B. (1982). Blood vessel fibronectin increases in conjunction with endothelial cell proliferation and capillary ingrowth during wound healing. *Invest. Dermatol.*, **79**, 269.

COOPER, A.R., KURKINEN, M., TAYLOR, A. & HOGAN, B.L.M. (1981). Studies on the biosynthesis of laminin by murine parietal endoderm cells. *Eur. J. Biochem.*, **119**, 189.

CURRAN, R.C. & GREGORY, J. (1978). Demonstration of immunoglobulin in cryostat and paraffin sections of human tonsil by immunofluorescence and immunoperoxidase techniques. *J. Clin. Pathol.*, **31**, 974.

DE LELLIS, R.A., STERNBERGER, L.A., MANN, R.B., BANKS, P.M. & NAKANE, P.K. (1979). Immunoperoxidase techniques in diagnostic pathology. *Am. J. Clin. Pathol.*, **71**, 483.

DUKES, C.E. (1932). The classification of cancer of the rectum. *J. Pathol. Bacteriol.*, **35**, 1489.

DVORAK, H.F., DICKERSIN, G.R., DVORAK, A.M., MANSEAU, E.J. & PYNE, K. (1981). Human breast carcinoma: fibrin deposits and desmoplasia: inflammatory cell type and distribution: microvasculature and infarction. *J. Natl Cancer Inst.*, **67**, 335.

EKBLOM, P., MIETTINEN, M., RAPOLA, J. & FOIDART, J.M. (1982). Demonstration of laminin, a basement membrane glycoprotein, in routinely processed formalin-fixed human tissues. *Histochemistry*, **75**, 301.

FINLEY, J.C.W. & PETRUSZ, P. (1982). The use of proteolytic enzymes for improved localization of tissue antigens with immunocytochemistry. In: *Techniques in Immunocytochemistry*. (Eds. Bullock & Petrusz), London: Academic Press, Vol. 1, p. 239.

HEATHCOTE, J.G. & GRANT, M.E. (1981). The molecular organization of basement membranes. *Int. Rev. Conn. Tiss. Res.*, **9**, 191.

HEIDENHAIN, M. (1908). Uber die haltbarkeit mikroskopischer praparate, insbesondere uber die Nachbehandlung jodierter gewebe mit natrium thiosulfate. *A. Wiss. Mikrosk.*, **xxv**, 397.

HOLUND, B., CLAUSEN, P.P. & CLEMMENSEN, I. (1981). The influence of fixation and tissue preparation on the immunohistochemical demonstration of fibronectin in human tissue. *Histochemistry*, **72**, 291.

HOLUND, B., CLEMMENSEN, I., JUNKER, P. & LYON, H. (1982). Fibronectin in experimental granulation tissue. *Acta. Pathol. Microbiol. Immunol. Scand.* Sect. A **90**, 159.

KLEINMAN, H.K., KLEBE, R.J. & MARTIN, G.R. (1981). Role of collagenous matrices in the adhesion and growth of cells. *J. Cell Biol.*, **88**, 473.

KUHL, U., TIMPL, R. & VONDER MARK, R. (1982). Synthesis of type IV collagen and laminin in cultures of skeletal muscle cells and their assembly on the surface of myotubes. *Develop. Biol.*, **93**, 344.

LINSENMAYER, T.F., FITCH, J.M., SANDERSON, R. & MAYNE, R. (1982). Monoclonal antibodies against collagen types IV and V. *J. Cell Biol.*, **95**, 116(a).

LIOTTA, L.A. (1982). Tumor extracellular matrix. *Lab. Invest.*, **47**, 112.

LIOTTA, L.A., TRYGGVASON, K., GARBISA, S., GEHRON ROBEY, P. & ABE, S. (1981). Partial purification and characterization of a neutral protease which cleaves type IV collagen. *Biochemistry*, **20**, 100.

LIOTTA, L.A., RAO, C.N. & BARSKY, S.H. (1983). Tumour invasion and the extracellular matrix. *Lab. Invest.*, **49**, 636.

McCABE, R.P. & EVANS, C.H. (1983). The regulatory role of extracellular proteases in tumour growth. *Survey Synth. Pathol. Res.*, **2**, 1.

MEPHAM, B.L., FRATER, W. & MITCHELL, B.S. (1979). The use of proteolytic enzymes to improve immunoglobulin staining by the PAP technique. *Histochem. J.*, **11**, 345.

MORSON, B.C. & DAWSON, I.M.P. (1979). *Gastrointestinal Pathology*, 2nd edition. Oxford: Blackwell, p. 656.

MULLINS, D.E. & ROHRLICH, S.T. (1983). The role of proteinases in cellular invasiveness. *Biochim. Biophys. Acta.*, **695**, 177.

NICOLSON, G.L. (1982). Cancer metastasis. Organ colonization and the cell-surface properties of malignant cells. *Biochim. Biophys. Acta*, **695**, 113.

NIEMCZUK, P., PERKINS, R.M., TALBOT, I.C. & CRITCHLEY, D.R. (1982). Lack of correlation between metastasis of human rectal carcinoma and the absence of stromal fibronectin. *Br. J. Cancer*, **45**, 500.

OHTANI, H. & SASANO, N. (1983). Stromal cell changes in human colorectal adenomas and carcinomas. *Virchows Arch.* [*Pathol. Anat.*] **401**, 209.

PAULI, B.U., SCHWARTZ, D.E., THONAR, E.J.M. & KUETTNER, K.E. (1983). Tumour invasion and host extracellular matrix. *Cancer Metastasis Rev.*, **2**, 129.

REPESH, L.A., FITZGERALD, T.J. & FURCHT, L.T. (1982). Fibronectin involvement in granulation tissue and wound healing in rabbits. *J. Histochem. Cytochem.*, **30**, 351.

SAINTE-MARIE, G. (1962). A paraffin embedding technique for studies employing immunofluorescence. *J. Histochem. Cytochem.*, **10**, 250.

SALO, T., LIOTTA, L.A., KESKI-OJA, J., TURPEENNIEMI-HUJANEN, T. & TRYGGVASON, K. (1982). Secretion of basement membrane collagen degrading enzyme and plasminogen activator by transformed cells – role in metastasis. *Int. J. Cancer*, **30**, 669.

SALO, T., LIOTTA, L.A. & TRYGGVASON, K. (1983). Purification and characterization of a murine basement membrane collagen-degrading enzyme secreted by metastatic tumour cells. *J. Biol. Chem.*, **258**, 3058.

SCOTT, D.L., MORRIS, C.J., BLAKE, A.E., LOW-BEER, T.S. & WALTON, K.W. (1981). Distribution of fibronectin in the rectal mucosa. *J. Clin. Pathol.*, **34**, 749.

SIEGAL, G.P., BARSKY, S.H., TERRANOVA, V.P. & LIOTTA, L.A. (1981). Stages of neoplastic transformation of human breast tissue as monitored by dissolution of basement membrane components. An immunoperoxidase study. *Invasion Metastasis*, **1**, 54.

STERNBERGER, L.A. (1979). *Immunocytochemistry* 2nd ed. John Wiley, New York. p. 124.

SZARFMAN, A., HASSELL, J.R., ROHRBACH, D.H., STANLEY, J.R. & MARTIN, G.R. (1982). Components of basement membranes: their properties, functions and alterations in disease states. In: *New Trends in Basement Membrane Research*. (Eds. Kuehn *et al.*), New York: Raven Press, p. 00.

TALBOT, I.C., RITCHIE, S., LEIGHTON, M.H., HUGHES, A.O., BUSSEY, H.J.R. & MORSON, B.C. (1980). The clinical significance of invasion of veins by rectal cancer. *Br. J. Surg.*, **67**, 439.

TALBOT, I.C., RITCHIE, S., LEIGHTON, M., HUGHES, A.O., BUSSEY, H.J.R. & MORSON, B.C. (1981). Invasion of veins by carcinoma of rectum: method of detection, histological features and significance. *Histopathology*, **5**, 141.

TERRANOVA, V.P., ROHRBACH, D.H. & MARTIN, G.R. (1980). Role of laminin in the attachment of PAM 212 (epithelial) cells to basement membrane collagen. *Cell*, **22**, 719.

TOWBIN, H., STAEHELIN, T. & GORDON, J. (1979). Electrophoretic transfer of proteins from polyacrylamide gels to nitrocellulose sheets: procedure and some applications. *Proc. Natl Acad. Sci.*, **76**, 4350.